# An evaluation of the emerging vaccines and immunotherapy against staphylococcal pneumonia in children

**DOI:** 10.1186/1471-2458-11-S3-S27

**Published:** 2011-04-13

**Authors:** Tanvir Huda, Harish Nair, Evropi Theodoratou, Lina Zgaga, Ali Fattom, Shams El Arifeen, Craig Rubens, Harry Campbell, Igor Rudan

**Affiliations:** 1 International Centre for Diarrhoeal Disease Research, Bangladesh (ICDDR,B), Dhaka, Bangladesh; 2 Centre for Population Health Sciences, Global Health Academy, The University of Edinburgh, UK; 3 Public Health Foundation of India, New Delhi, India; 4 Nabi Biopharmaceuticals, Rockville, MD, USA; 5 Global Alliance to Prevent Prematurity and Stillbirth, Seattle Children's, Seattle, WA, USA; 6 Croatian Centre for Global Health, University of Split Medical School, Croatia

## Abstract

**Background:**

Staphylococcus aureus is a commensal of human skin and nares. It is also one of the leading nosocomial pathogens in both developed and developing countries and is responsible for a wide range of life threatening infections, especially in patients who are immunocompromised, post-surgery, undergoing haemodialysis and those who are treated with catheters and ventilators. Over the past two decades, the incidence of nosocomial staphylococcal infections has increased dramatically. Currently there are at least seven vaccine and immunotherapy candidates against S. aureus in the developmental phase targeting both active and passive immunization.

**Methods:**

We used a modified CHNRI methodology for setting priorities in health research investments. This was done in two stages. In Stage I, we systematically reviewed the literature related to emerging vaccines against Staphylococcus aureus relevant to several criteria of interest: answerability; cost of development, production and implementation; efficacy and effectiveness; deliverability, affordability and sustainability; maximum potential impact on disease burden reduction; acceptability to the end users and health workers; and effect on equity. In Stage II, we conducted an expert opinion exercise by inviting 20 experts (leading basic scientists, international public health researchers, international policy makers and representatives of pharmaceutical companies) to participate. The policy makers and industry representatives accepted our invitation on the condition of anonymity, due to sensitive nature of their involvement in such exercises. They answered questions from CHNRI framework and their “collective optimism” towards each criterion was documented on a scale from 0 to 100%.

**Results:**

The panel of experts expressed low levels of optimism (score around or below 50%) on the criteria of answerability, efficacy, maximum disease burden reduction potential, low cost of production, low cost of implementation and affordability; moderate levels of optimism (scores around 60 to 80%) that these vaccines could be developed at a low cost, and thus on the deliverability, sustainability and impact on equity; and high levels of optimism (scores above 80%) regarding acceptable of such a product to both the end-users and health workers. While assessing the candidates for passive immunization against S.aureus, the experts were poorly optimistic regarding low production cost, low implementation cost, efficacy, deliverability, sustainability, affordability and equity; moderately optimistic regarding answerability and acceptability to health workers and end-users. They were of the opinion that these interventions would have only a modest impact (3 to 5%) on the burden of childhood pneumonia. .

**Conclusion:**

In order to provide an effective vaccine against *S. aureus*, a number of unresolved issues in vaccine development relating to optimal antigenic target identification, criteria for acceptable efficacy, identification of target population, commercial development limitations, optimal timing of immunization strategy, storage, cold chain requirements and cost need to be addressed properly. There is still a great deal unknown about the complex interaction between *S. aureus* and the human host. However, given the nature of *S. aureus* and the lessons learned from the recent failure of two emerging vaccines, it is clear that a multi-component vaccine is essential. Combating only one virulence factor is not sufficient in the human host but finding the right combination of factors will be very challenging.

## Background

Pneumonia is the leading cause of global child mortality. Approximately 1.6 million children under the age of 5 years die each year due to pneumonia [1]. Most prospective aetiology studies of pneumonia suggest that *Streptococcus pneumoniae* (pneumococcus) and *Haemophilus influenzae type b* (HiB) are the leading bacterial causes followed by *Staphylococcus aureus* (Staphylococcus) and *Klebsiella pneumoniae*. Though effective vaccines exist against the two major causes of bacterial pneumonia, no vaccine is presently available against *S. aureus*.

*Staphylococcus aureus* is a Gram-positive bacterial commensal of human skin and nares*.* About 20-30% of the human population are *S. aureus* carriers and show little resistance to mucosal colonization by the pathogen [2,3]. Colonization may be transient or persistent and can last for years [4]. *Staphylococcus aureus* is also one of the leading nosocomial pathogens in both developed and developing countries, causing infection frequently in immunocompromised patients, surgical patients, patients undergoing haemodialysis and those who are treated with catheters and ventilators [2]. In the past 20 years the incidence of nosocomial staphylococcal infections has increased dramatically. It is now responsible for approximately 25% of the 2 million nosocomial infections reported in the United States each year [5]. In addition, the increasing trend of *methicillin-resistant S. aureus* (MRSA) infection has posed new problems. MRSA is now endemic in hospitals around the world with an estimated 1.5 million cases per year worldwide [6,7]. The incidence of community-acquired MRSA infections are also increasing and there are reports of MRSA strains with reduced susceptibility to Vancomycin [8-11]. This establishes a need for new treatment and prevention strategies against *S. aureus*.

Vaccine development needs extensive research and resources. The development of Staphylococcal vaccine is further complicated by the pleomorphic character of staphylococci and complex patient populations at risk. The target population for *S. aureus* vaccination is different from other vaccines against pneumonia. In order to provide a full range of protection both active and passive immunization approaches need to be taken. An active immunization strategy may be a feasible approach for preventing staphylococcal infections in immunocompetent patients scheduled to undergo elective procedures. Populations at high risk for *S. aureus* infections, where active immunization is unlikely to be helpful, include neonates, especially premature newborns; other more completely immunocompromised children (e.g., certain cancer patients on immunosuppressive therapy); and populations where the risk of infection is both high and immediate (e.g., shock-trauma patients). One approach to providing these individuals with immunoprophylaxis is to use these vaccines as immunizing agents in healthy adult plasma donors, collect their plasma, and then fractionate it to produce specific hyper-immune gamma immunoglobulin (IGIV) for intravenous passive immunization. It should be noted that for some patients, such as those receiving prosthetic devices e.g. hip replacements, it may be necessary to provide both passive and active immunization in order to protect the individual from infection immediately after surgery and in the longer term.

It is therefore very important to assess the potential impact of all emerging vaccines and immunotherapy against *Staphylococcus aureus* and determine an investment strategy based on key prioritization factors. Currently there are at least seven products against *S. aureus* in the developmental phase targeting both active and passive immunization. We aimed to review the existing literature, outlining the progress of the emerging vaccines and immunotherapy against *Staphylococcus aureus* at all stages of development; present the evidence regarding key issues surrounding these products and assess the level of collective optimism of international experts over their priority status for receiving investment support. The paper is presented as part of a series of papers, each in turn focusing on different emerging vaccines and other interventions against pneumonia.

## Methodology

We used a modified Child Health and Nutrition Research Initiative (CHNRI) methodology for setting priorities in health research investments. The methodology has been described in great detail [12-16] and implemented in a variety of settings [16-22].

### CHNRI exercise – stage I: identification and selection of studies

**Figure 1:**
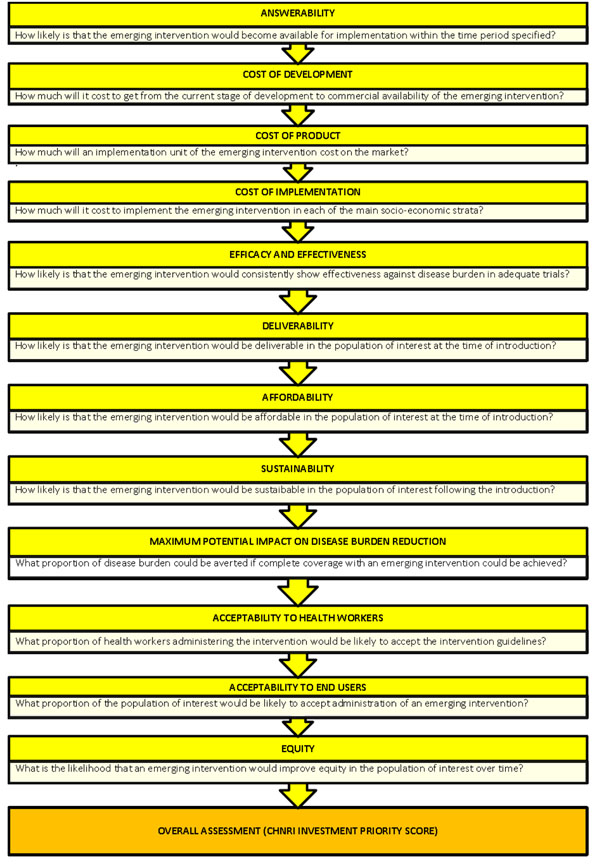
**A summary of Stage I of the CHNRI process of evaluation of an emerging intervention (a systematic review of the key CHNRI criteria)**. CHNRI- Child Health and Nutrition Research Initiative

We conducted a systematic literature review using the following criteria: answerability, cost of development, cost of product, cost of implementation, efficacy and effectiveness, deliverability, affordability, sustainability, maximum potential impact on disease burden reduction, acceptability to health workers, acceptability to end users and equity [19] (Figure 1). Searches were conducted initially in July 2009 (and updated in April 2010) and were limited to Ovid MEDLINE, Web of Knowledge, Google Scholar and Cochrane central register for controlled trials. No language or publication restrictions were applied. In order to ensure completeness, we also conducted hand searching of online journals, scanned the reference list of identified citations, and checked literature available on the websites of pharmaceutical companies (Inhibitex Inc., Merck, Nabi Biopharmaceuticals, Neutec Pharma Ltd. and Biosynexus) and international agencies (GAVI, WHO, UNICEF and Pneumo ADIP). Details of the search strategies used are presented in Additional file 1.

### CHNRI exercise – stage II: an expert opinion exercise

**Figure 2:**
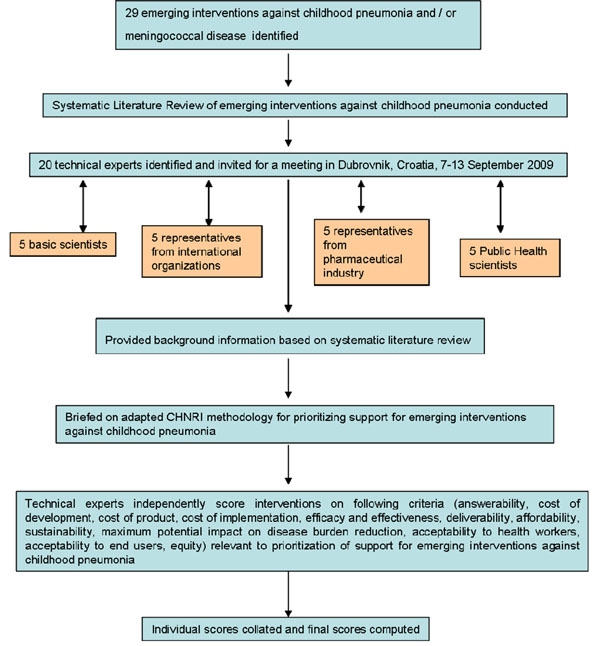
**A summary of Stage II of the CHNRI process of evaluation of an emerging intervention (an expert opinion exercise using the CHNRI criteria).** CHNRI- Child Health and Nutrition Research Initiative

We shared the initial review of the literature with 20 experts. The list of chosen experts included five leading basic scientists, five international public health researchers, five international policy makers and five representatives of the pharmaceutical companies. The 20 experts were chosen based on their excellent track record in child health research (but were not specifically involved with staphylococcal disease research). We initially offered participation to the 20 experts with the highest impact publications in their area of expertise over the past 5 years (for basic researchers and international public health researchers), or to individuals who were affiliated with pharmaceutical companies that had large vaccination programmes or working in large-budget international agencies. For those who declined to participate (about 20%) replacements were found using the same criteria. The policy makers and industry representatives accepted our invitation on the condition of anonymity, due to sensitive nature of their involvement in such exercises. About half of the experts were either affiliated to institutions in developing countries or had previous experience of working in developing country settings. The experts met during September 7-13, 2009 in Dubrovnik, Croatia, to conduct the 2^nd^ stage of CHNRI expert opinion exercise. The process of second-stage CHNRI is shown in Figure 2. All invited experts discussed the evidence provided in CHNRI stage I, and then answered questions from the CHNRI framework (Supplementary table 2 in additional file 1). Their answers could have been “Yes” (1 point), “No” (0 points), “Neither Yes nor No” (0.5 points) or “Don’t know” (blank). Their “collective optimism” towards each criterion was documented on a scale from 0 to 100%. The interpretation of this metric for each criterion is simple: it is calculated as the number of points that each evaluated type of emerging intervention against *Staphylococcus aureus* received from 20 experts (based on their responses to questions from the CHNRI framework), divided by the maximum possible number of points (if all answers from all experts are “Yes”). [12-16].

## Results

**Figure 3:**
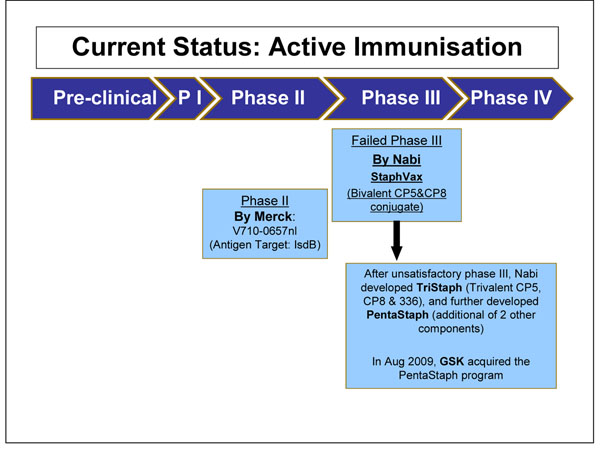
The current status of the research into Staphylococcal vaccines presented to the expert group for stage II of the CHNRI process

**Figure 4:**
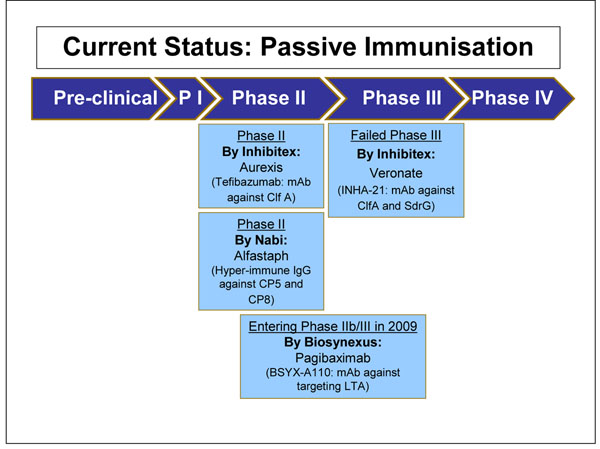
The current status of the research into passive immunization against *S. aureus* presented to the expert group for stage II of the CHNRI process

We identified 63 articles and product monographs for inclusion. Several products are currently in development phase, most of which have completed phase I and II clinical trials (Figure 3 and Figure 4). Each product targets different virulence factors of the *S. aureus* pathogen. These factors include Staphylococcal surface proteins, polysaccharides, exoproteins and toxins elaborated by *S. aureus*. The only product which completed a Phase III clinical trial is Aurograb. Aurograb was a human-derived single chain variable fragment (scFv) therapeutic antibody against the *S. aureus* ATP-binding cassette (ABC) transporter. Aurograb was developed for the treatment of deep-seated MRSA infections. In 2006, the product completed a double-blind placebo-controlled phase III clinical trial carried out in a total of 35 centres in 6 European countries. However in 2008 the company decided not to pursue further development of Aurograb. The potential impact of Aurograb will not be assessed in this paper.

### Answerabilty

#### Active immunization

##### CP5 /CP8 Conjugate vaccines (StaphVAX, PentaStaph)

Bacterial capsular polysaccharides (CPs) confer resistance against host immune mechanisms and promote virulence. Antibodies to these CPs were shown to protect against infections caused by *pneumococci*, *meningococci*, *Haemophilus influenzae type b* and other pathogens. *S. aureus* isolates were initially believed not to possess capsular polysaccharides. However, Karakawa and colleagues discovered that S. aureus clinical isolates are capsulated and similar to *pneumococci* and *group b Streptococci* in that they posses several different capsular types [23]. They also showed that conjugate vaccines to these polysaccharides generate antibodies that mediate type-specific opsonophagocytosis in an in vitro opsono-phagocytic assay that contained complement and neutrophils [24]. Of the 13 known serotypes, two capsular types, 5 and 8, are the most important as they comprise the majority (~85%) of clinical isolates [9,25,26]. Fattom and colleagues combined the capsular polysaccharide 5 and capsular polysaccharide 8 to the mutant non toxic recombinant Pseudomonas aeruginosa exotoxin A (rPEA) and formed the bivalent vaccine StaphVAX [27].

##### Iron regulated surface determinant B (V710)

The potential of surface proteins of gram positive bacteria as an antigen has been tested for many years. However, in the case of *S. aureus*, none of the surface proteins that were tested as an antigen were found to be essential components of the pathogen. There is a high level of redundancy in the virulence protein range. Such redundancy makes the loss of a specific protein non fatal in *S. aureus*. Iron regulated surface determinant B (IsdB), an iron-sequestering protein, is conserved in diverse *S. aureus* clinical isolates, both methicillin resistant and methicillin sensitive. IsdB is expressed when there is iron limitation and has a role in the acquisition of iron [28]. IsdB was first identified as a candidate antigen by Etz and colleagues [29]. It has been reported that although IsdB is not an essential protein for *S. aureus* in vitro and loss of this protein results in a reduction in virulence in vivo, which makes it an attractive vaccine candidate [28]. Merck`s new vaccine V710 contains IsdB protein antigen.

**Figure 5:**
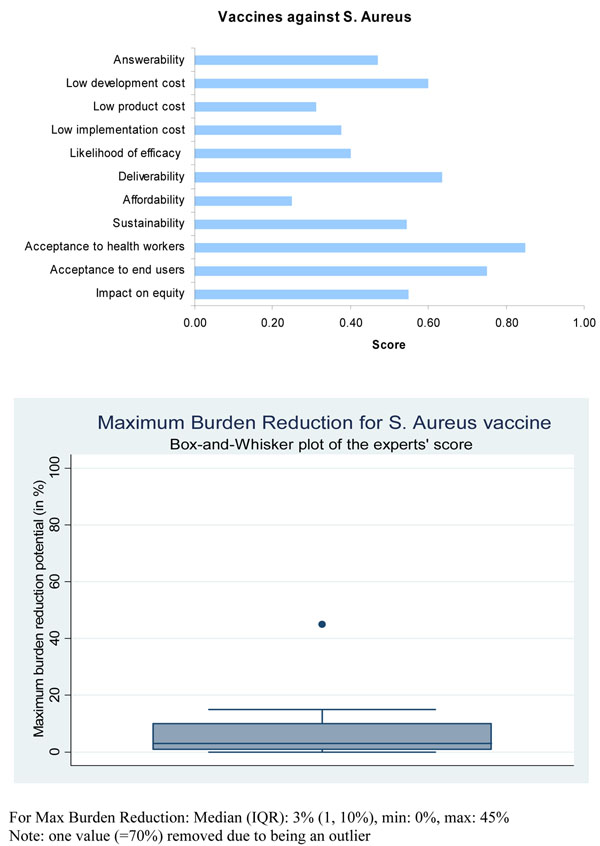
**The results of Stage II CHNRI process – an expert opinion exercise assessing the potential usefulness of investment in staphylococcal vaccines**. CHNRI- Child Health and Nutrition Research Initiative

Presented with this evidence, the panel of experts expressed a low level of optimism (score around 40%) regarding the ability of vaccines for active immunization against *S. aureus* to satisfy the criterion of answerability (Figure 5).

#### Passive immunization approach

##### S. aureus Clumping factor A- based vaccines (Veronate and Aurexis )

*Staphylococcus aureus* colonizes the host by adhering to components of the extra cellular matrix through surface protein adhesions known as microbial surface components recognizing adhesive matrix molecules (MSCRAMM) [30]. MSCRAMM bind to extracellular matrix components within host tissues or to serum-conditioned implanted biomaterials (e.g catheters, artificial joints, and vascular grafts). Evidence suggests that this microbial adherence is an important factor in the initiation and metastatic spread of *S. aureus* infections [31]. Therefore, interventions that impact on early events in the infectious process may lead to an improved clinical outcome. MSCRAMMs have therefore been targeted as potential antigenic candidates for the development of novel immunotherapies. One such MSCRAMM protein is Clumping factor A (ClfA), an adhesin that mediates *S. aureus* binding to fibrinogen. It is expressed on the surface of almost all strains of *S. aureus*[8]. ClfA recognizes the C terminus of the γ chain of human fibrinogen [29,30,32] and antibodies raised against the A domain of ClfA can inhibit the interaction between ClfA and fibrinogen. Based on this, Inhibitex developed INH-A21 (Veronate), which is a human intravenous immune preparation derived from donors with high levels of antibodies against the staphylococcal fibrinogen binding proteins ClfA and Ser-Asp dipeptide repeat G (SdrG).

Another similar product in the pipeline that is also being developed for the treatment of serious *S. aureus* bacteremia and related complications is Tefibazumab (Aurexis) [33]. This is a humanized immunoglobulin G1 monoclonal antibody that specifically recognizes ClfA with a high affinity.

##### Hyper immunoglobulin from patients immunuized with CP5 /CP8 Conjugate vaccines (AltaStaph)

A parallel track for developing immunotherapy against *S. aureus* was developed concurrently to the development of the StaphVAX. This approach utilized the vaccine to produce hyper-immune IgG for the treatment of patients at high risk for *S. aureus* infections that could not mount a significant immune response to the vaccine, or to be used as an adjunct therapy in patients who are already infected with *S. aureus*. Plasma donors were immunized once with StaphVAX and plasma were collected at least once a week starting two weeks after immunization. Plasma pools were made and IgG was fractionated using the Cohn fractionation technique. The purified IgG was formulated as intravenous immunoglobulin preparation (IVIG) at 50mg/ml IgG. The types 5 and 8 CP antibodies comprised up to 6% of the total IgG.

##### Chimeric monoclonal antibody (Pagibaximab)

Lipoteichoic acid (LTA) is a major constituent of the cell wall of gram positive bacteria and consists of teichoic acids, which are long chains of ribitol phosphate and glycerolipid. Available evidence suggests that LTA possess antigenic properties. Biosynexus has developed a humanized mouse chimeric mAB agaistt LTA called pagibaximab (BSYX – A110). The antibody is targeted at low birth weight infants for the prevention of blood stream infections by *S. aureus* and coagulase negative staphylococci [34].

**Figure 6:**
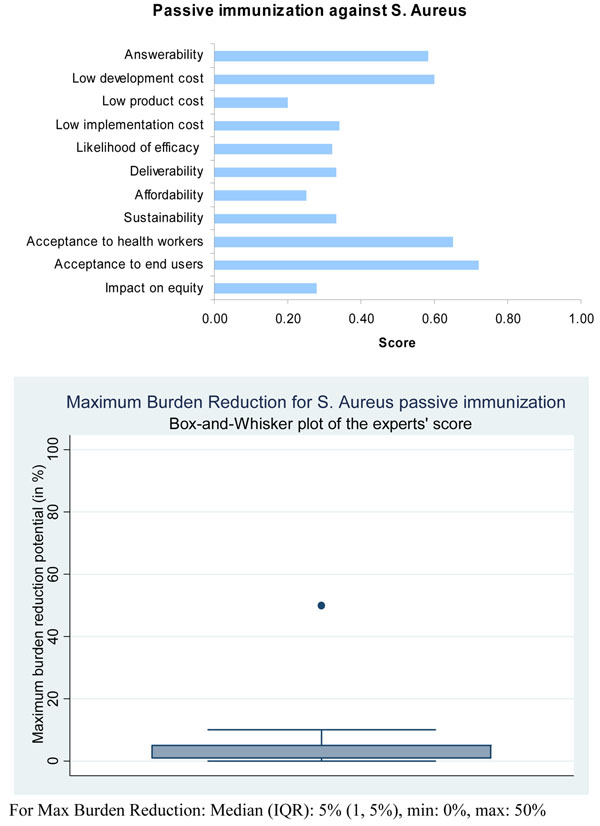
**The results of Stage II CHNRI process – an expert opinion exercise assessing the potential usefulness of investment in passive immunization against *S.aureus.*** CHNRI- Child Health and Nutrition Research Initiative

Although not supported by hard evidence, the panel of experts expressed moderate levels of optimism (median score around 60 percent, which was greater than for active immunization) concerning the ability of immunotherapy for passive immunization to satisfy the criterion of answerability (Figure 6).

### Efficacy and effectiveness

#### Active immunization

##### CP5 /CP8 Conjugate vaccines (StaphVAX, TriStaph, PentaStaph)

The first phase III clinical study of the StaphVAX vaccine conducted on 1804 haemodialysis patients showed mixed results [35]. Although the study population comprised of extremely immunocompromised patients, the vaccine elicited antibody response levels of at least 80 µg per millilitre (the estimated minimal protective level) in 80% of patients for CP5 and in 75% of patients for CP8. However, the efficacy was not sustained. The efficacy during weeks 3 to 54 was only 26%.

This study confirmed, for the first time, the concept that capsular antibodies can afford protection against *S. aureus* infections. However, in these severely immuno-compromised patients a different regimen that can sustain high levels of antibodies beyond one year is needed. A confirmatory Phase III clinical trial with 3600 haemodialysis patients was conducted and included a booster immunization at 8 months following the first immunization. The confirmatory phase III study reported no significant protection against *S. aureus*. The failure was attributed to slight changes in manufacturing that resulted in a suboptimal antibodies generated by the vaccine. A press release by Nabi (http://www.nabi.com) dated 21 March 2006 stated : “The quality or functional characteristics of the antibodies generated by the vaccine used in the confirmatory clinical study was inferior to those antibodies generated by vaccine lots used in previous and subsequent clinical studies”. We found no report on further study of StaphVAX.

The clinical outcome from these studies and other animal model studies suggests that capsular polysaccharides are appropriate for generating protective opsonic antibodies. However, more antigens are need to be added to extend the coverage of the vaccine and to neutralize significant toxins that debilitate host immune competency.

Nabi added three more components to their original StaphVAX formula to form the five component vaccine PentaStaph [36,37]. The new surface polysaccharide component, 336, induces antibodies against Type 336 cell wall antigen while the other two candidate component generates antibodies that neutralize Panthon Valentine Leukocydin, PVL, a unique leukocidin toxin, produced by the CA-MRSA strains and alpha toxin produced by almost all *S. aureus* isolates; both these debilitate host immune competency including the opsonophagocytosis process.

In August 2009 GSK acquired the PentaStaph program from Nabi and are currently undertaking its further development. [http://www.nabi.com/pipeline]

##### Iron regulated surface determinant B (V710)

The vaccine was found to be immunogenic in an animal model when it was formulated with amorphous aluminum hydroxyphosphate sulfate adjuvant. Three phase I studies were conducted to evaluate different formulations (Liquid Aluminum –adjuvanted, Liquid non adjuvanted, and lyophilized) of V710 vaccine [28,38]. The results of the studies showed similar immunogenecity with all formulations. A positive immune response, as defined by more than a two fold increase in antibody levels, ranges from 72% to 84% across different formulations. No serious side effects or fever were reported. The most common vaccine related adverse effects were pain at injection site and headache [38]. Phase II clinical trials are currently underway to evaluate the efficacy and safety of a single dose of the vaccine in patients undergoing elective cardiothoracic surgery and to assess the safety and immunogenicity of the vaccine candidate in patients with end-stage kidney disease who are receiving hemodialysis. A randomized, multicenter, double-blind, group-sequential study to evaluate the efficacy, immunogenicity, and safety of a single dose of V710 in adult patients scheduled for cardiothoracic surgery is currently underway (http://clinicaltrials.gov/ct2/show/NCT00518687). It is too early to judge the efficacy of the new vaccine.

Based on this limited evidence, the panel of experts expressed a relatively low level of optimism (score around 40%) concerning the likelihood of efficacy of vaccines for active immunization against *S. aureus* to reduce childhood pneumonia mortality (Figure 5).

#### Passive immunization

##### S. aureus Clumping factor A- based vaccines (Veronate and Aurexis )

A multicenter double blind study was conducted in infants with very low birth weight to assess the safety profile and efficacy of INH-A21[39]. Infants were randomized to three different dose groups (250, 500 or 750 mg/kg). The INH-A21 750 mg/kg group (N = 157) reported fewer episodes of *Staphylococcus aureus* sepsis [RR=0.37; P = 0.14], candidemia (RR=0.34; P = 0.09) and mortality (RR=0.64; P = 0.27) when compared with the placebo-treated cohort (N = 158). A follow up phase III double blinded placebo controlled study [40] was conducted with 1983 infants to retest the safety and efficacy of INH-A21. The primary outcome measure was the rate of *S. aureus* associated late-onset sepsis (LOS) which developed in 5% and 6% of infants who received the placebo or INH-A21 respectively (P = 0.34). Disappointingly, no differences were found in the frequencies of LOS, candidemia, or overall mortality in the two groups. However, Schaffer and Lee in their review of vaccines against *S.aureus* suggested [8] that as the INH-A21 product was not elicited by immunization but instead by natural exposure to staphylococci, it is possible that the antibodies might have recognised the wrong ClfA epitopes or may have been of low affinity or avidity towards their target antigens. An adequately powered, well-controlled study was recommended to further assess the efficacy and safety of INH-A21.

A randomized, double-blind, placebo-controlled, multicenter phase II clinical trial was conducted in hospitalised patients with documented *S. aureus* bacteraemia [29] to evaluate the efficacy of Tefibazumab (Aurexis). The efficacy was assessed in terms of relapse of *S. aureus* bacteraemia (SAB), complications related to *S. aureus* bacteraemia, or death. Two of 30 (6.7%) patients reached the composite clinical endpoint in the Tefibazumab group while for the placebo group the number was 4 out of 30 (13.3%) (P = 0.455). However, several limitations in this study were reported with respect to outcomes. There were differences in baseline characteristics of the study populations and the use of antibiotic in the protocol was not standardized. Moreover, adjunctive treatments, such as surgery and timing of catheter removal, were not taken into consideration in the study analysis. The most frequently reported adverse events were hypokalemia, diarrhea, anemia, and insomnia but there were no significant differences across the treatment groups. As expected for a group of patients with SAB, twelve (40%) patients in the Tefibazumab group and nine (30%) patients in the placebo group had at least one serious adverse event. The safety data from this clinical trial and preliminary clinical data support continued clinical development. More trials are needed to evaluate efficacy of this product.

##### Hyper immunoglobulin from patients immunized with CP5 /CP8 Conjugate vaccines (AltaStaph)

A multicenter Phase 2 clinical trial to assess the safety and pharmacokinetics of AltaStaph in very low birth weight (VLBW) infants was performed. Neonates were infused twice in two weekly intervals with saline or AltaStaph at 1000mg/kg. Levels for CPS types 5 before the second infusion were 188 mcg/ml. Type 8 IgG levels were similar. Geometric mean IgG levels among placebo recipients were consistently <2 and <5 mcg/ml for types 5 and 8 respectively in both weight groups. The conclusion from this study was that infusion of AltaStaph in VLBW neonates resulted in high levels of specific *S. aureus* types 5 and 8 CPS IgG. The administration of this anti-staphylococcal hyperimmune globulin was well tolerated in this population [41]. Since the study was not powered to detect clinical outcomes, the data generated did not show differences in the rate of *S. aureus* infection between the two study arms.

In another study in adults with culture proven bacteremia, AltaStaph was evaluated as a potential adjunctive therapeutic in adult subjects with *S. aureus* bacteremia and persistent fever. Administration of AltaStaph had a positive effect on the APACHE II scores of these patients compared to patients who received the placebo treatment. This was further supported by the time to hospital discharge. For all subjects, the median time to discharge excluding deaths that occurred during hospitalization, which could bias the results, were 14 and 9 days in the placebo and AltaStaph groups respectively (P=0.033). It was concluded that AltaStaph was shown to be well tolerated in subjects with *S. aureus* bacteremia and warranted further evaluation as an adjunct therapy against multidrug resisitant *S. aureus* bacteremia [42].

##### Chimeric monoclonal antibody (Pagibaximab)

A phase I/II dose escalation, safety and pharmacokinetics study of Pagibaximab was conducted and the findings showed no difference in morbidities and mortality across study groups. All serious adverse effects were deemed unrelated or probably not related to the drug. However, no evidence of a response to Pagibaximab was detected [43]. Another phase II randomized double-blind study of Pagibaximab in very low birth weight neonates concluded that three infusions of Pagibaximab 60 or 90 mg/kg, administered 1 week apart to high-risk neonates, appeared safe, well tolerated, demonstrated linear pharmacokinetics, and at 90 mg/kg produced potentially protective levels of antibody [44].

Based on these evidence, the panel expressed a low level of optimism (score around 30 percent) regarding the likelihood of efficacy of immunotherapy for passive immunization against *S. aureus.*

### Maximum potential for disease burden reduction

Rudan et al. [45] estimated that the incidence of clinical pneumonia in under 5 children in developing countries was about 0.29 episodes per child year or 151.8 million new cases every year. In contrast, the number of new cases per year in developed countries was estimated to be around 4 million. The Child Health Epidemiology Reference Group (CHERG) estimated that around 1.6 million deaths in under five children were attributable to pneumonia in 2008 [1].

Results from prospective microbiology-based pneumonia etiology studies revealed that the leading bacterial cause for pneumonia is pneumococcus (isolated in 30–50% of pneumonia cases) while the second most common isolated pathogen was *H. influenzae* type b. *Staphylococcus aureus* was identified as the third most common bacterial cause [46-53]. A study in Chile using lung aspirate cultures found *S. aureus* to be the main pathogen [54]. Another WHO study of hospitalised children with very severe pneumonia in seven countries found *S. aureus* in 42% of cases making it the second largest cause [32]. Studies of nosocomial pneumonia and ventilator associated pneumonia have demonstrated that *S. aureus* was responsible for majority of the cases [55,56]. Furthermore, MRSA has been identified as one of the most common pathogens in all forms of pneumonia [57]. In recent years, the HIV epidemic has also led to an increase in the incidence and mortality from childhood pneumonia. Although there are limited data on the causes of neonatal pneumonia in developing countries, studies on the aetiology of neonatal sepsis suggest Klebsiella spp., Group B Streptococcus and *S. aureus* are the main causes of neonatal pneumonia [58].

Developing an effective vaccine would result in a significant reduction of disease burden from *S. aureus* infections. However, quantification of the maximum reduction of disease burden using the staphylococcus vaccines without any information on the burden of disease from *S. aureus* pneumonia and studies on vaccine effectiveness is not possible. None of the vaccine candidates have passed phase III trials. The major problem with developing staphylococcal vaccines is the lack of understanding of how virulence factors or potential antigens are expressed by bacteria in the host versus those that have been studied in vitro. However, given the pleomorphic nature of the pathogen and the failure of single component vaccines, it is anticipated that without a multi-component vaccine a significant reduction of disease burden will not be possible [8].

The panel was of the opinion that both types of immunizations against *S. aureus* were likely to have a relatively low level of maximum impact (3 to 5 percent) on disease burden due to childhood pneumonia (Figure 5 and Figure 6).

### Deliverability, affordability, sustainability and cost

Deliverability and sustainability of any new vaccine strategy depends on the infrastructure and resources required to deliver the vaccine. Most of the vaccines need cold storage facilities. In many developing countries the cold chain system is breaking down. A study in Ethiopia found 31% of the equipment to be non-functional, with a large number of items having exceeded the manufacturer’s recommended working life [59]. So a heat stable *S. aureus* vaccine could achieve a high coverage even in the hard to reach areas of the developing countries. However, we were unable to find any reports which would suggest that the candidate vaccines presently under development are using technology that can retain the potency and efficacy at elevated temperatures. Storage is another key issue which needs to be taken into consideration while designing any vaccination program. The newer vaccines are often single-dose presentation in pre-filled glass syringes and bulky packaging which need more storage space. Although none of the staphylococcal vaccines are ready for licensure at this point of time, manufacturers will need to take these factors into consideration while designing the packaging for the newer vaccines.

As discussed earlier, an effective staphylococcal vaccination strategy will require quite a different immunization program and this is likely to increase the complexity of vaccine delivery mechanism in developing countries. Some have advocated that if an effective vaccine is developed it should be used in all children to prevent any type of staphylococcal disease. The recent increase in community-associated MRSA in children with no predisposing risk factors e.g. healthy newborns and young adults, and increase in the prevalence of *S. aureus* colonization in the general population support such argument [60]. However, it will remain a challenge to develop a staphylococcal vaccine that would be cross protective against multiple strains as well as strains which are of the same serotype but express antigens differently under the same conditions.

Another important factor determining the deliverability of a vaccine is cost. However, different initiatives have emerged recently to help the uptake of newer vaccines by developing countries. In 2007, WHO and PATH, with the support of the Bill & Melinda Gates Foundation, launched Optimize – a global effort to help countries manage immunization logistics. The International Finance Facility for Immunisation (IFFIm), a multilateral development institution, has been created to accelerate the availability of predictable long term funds for health and immunization programmes through the GAVI Alliance in 70 of the poorest countries in the world. Another similar initiative is Advance Market Commitment (AMC). Established in 2005 by the Center for Global Development and carried forward by five bilateral donor governments, the Bill & Melinda Gates Foundation, the GAVI Alliance, and the World Bank, the AMC aims to stimulate the development and manufacture of vaccines especially suited to developing countries. With the help of these initiatives it is hoped that the time delay between the introduction of new vaccines into developed and developing countries can be reduced.

Based on all available evidence the expert group expressed a moderate level of optimism (score about 60%) in the ability to develop a low cost intervention for active and passive immunizations against staphylococcus (Figure 5 and Figure 6). However, they expressed low levels of optimism (scores below 40%) that these interventions would have low production and implementation costs, which would have a direct impact on affordability, deliverability (more for passive immunization) and sustainability.

### Acceptability and equity

While assessing the impact of a new intervention on child health equity, the panel considered the following questions: given the present distribution of the disease burden from *S. aureus* infections, will the intervention be accessible to the underprivileged in the population and would it benefit them? And does the proposed research have the overall potential to improve equity in disease burden distribution in the long term?

Little information is available regarding the burden of disease from *S. aureus* infections in developing countries. It is therefore difficult to assess whether a staphylococcal vaccine will have a relatively greater impact on the poorest communities in the world. Studies which include new molecular techniques are required to provide a better knowledge base about the burden of disease from staphylococcus pneumonia in developing countries.

The panel of experts expressed a moderate level of optimism (score about 60 percent) over the impact of staphylococcal vaccines on equity compared to interventions for passive immunization (score about 40 percent). However, the panel was very optimistic (scores above 80%) that if such a vaccine were to be developed it would be acceptable to both end-users and health workers (Figure 5). They were only moderately optimistic (scores about 70%) regarding the acceptability of passive immunization against *S.aureus* to both health workers and end-users.

## Discussion

The literature review summarized in this paper presents evidence required for making an informed decision on the research priorities that should be given to emerging interventions against *S. aureus*. The scores for both active and passive immunization interventions against the set criteria represent the collective optimism of a panel of experts drawn from varying technical backgrounds and affiliations. Although several *S. aureus* vaccine candidates are currently in pre-clinical and clinical phases of development, none have yet been approved for licensure. The development of *S. aureus* vaccines has the potential to reduce the global burden of Staphylococcal pneumonia. However, a number of unresolved issues in *S. aureus* vaccine development need to be addressed properly in order to develop a successful vaccination strategy [8]. There is little evidence that supports the argument that immunity to *S. aureus* infection does indeed exist. It is also clear that *S. aureus* has developed the capability to defend itself against human innate immunity [61]. Imperfect animal models and the recent failure of two candidate vaccines at Phase III clinical trials has made the development process more risky and complicated.

While the collective optimism of the panel regarding vaccines against *S. aureus* on the CHNRI criteria was low on answerability, efficacy, maximum disease burden reduction potential, low cost of production, low cost of implementation and affordability; they were moderately optimistic that these vaccines could be developed at a low cost, and thus on the deliverability, sustainability and impact on equity; and highly optimistic that if such an intervention were to be developed, it would be highly acceptable to the end-users and health workers. While assessing the candidates for passive immunization against S.aureus, the experts were poorly optimistic regarding low production cost, low implementation cost, efficacy, deliverability, sustainability, affordability and equity; moderately optimistic regarding answerability and acceptability to health workers and end-users. They were of the opinion that these interventions would have only a modest impact (3 to 5%) on the burden of childhood pneumonia.

This is the first time that such an exercise has been conducted with the aim of predicting the future impact of emerging vaccines on morbidity and mortality due to childhood pneumonia. The CHNRI methodology was primarily designed to evaluate existing interventions and competing investment priorities for health research. Although we used the CHNRI set of criteria, we modified it by including a systematic review of available literature and not involving all stakeholders (e.g. end-users and health workers). The scores reported in this paper express the collective opinion of a panel of 20 experts. While there is always an element of error while predicting the impact of interventions which do not exist and have no clinical trial data to support them, we feel that the results would be reproducible with another panel in a different setting.

## Conclusions

To summarize, while it is not only important that investments are made in researching new vaccines, adequate emphasis must be made and resources allocated for proper distribution of the vaccine. Additionally, there are issues relating to optimal antigenic target identification, criteria for acceptable efficacy, identification of the target population in children as well as adults, commercial development limitations, optimal timing of immunization strategy, storage and cold chain requirements, cost of development and cost effectiveness (64). There is still a great deal unknown about the complex interaction between *S. aureus* and the human host. All vaccine candidates that have been tested so far were found to play important roles in vivo. However, none of these candidates have been found to be essential and this raises the concern that making vaccines against antigens that are not expressed in the host might lead to ineffective vaccines. Given the nature of *S. aureus* and the lessons learnt from the recent failure with two emerging vaccines, it is clear that a multi-component vaccine is essential. Combating only one virulence factor is not sufficient in the human host but finding the right combination of factors will be very challenging.

## Competing interests

TH, HN, ET, LZ, CR, HC and IR declare that they have no competing interests. At the time of writing this paper AF was employed by Nabi Biopharmaceuticals which is involved in the development of products against *Staphylococcus aureus* (subsequently acquired by GSK).

## Authors’ contributions

TH participated in the design of the study, led the literature review, data collection, data analysis, data interpretation and prepared the initial draft of the manuscript. HN participated in the design of the study, contributed to data collection and preparation of the revised manuscript. ET participated in design of the study, data collection, statistical analysis, data interpretation and critically reviewed the manuscript. LZ participated in the design of the study, data collection, data collection and critically reviewed the manuscript. AF and CR contributed to data interpretation and critical review of the manuscript. IR and HC conceived of the study, participated in literature review, data collection, data interpretation, and critically reviewed drafts of the manuscript. All authors read and approved the final manuscript.

## Supplementary Material

Additional file 1 Details of search strategy for identifying studies reporting novel interventions against *Staphylococcus aureus*.
Supplementary table 1: Details of search strategy for identifying studies reporting novel interventions against Staphylococcus aureus.
Supplementary table 2: Questions used in the phase II CHNRI process. Click here for file
